# Urinary incontinence (UI) in older women in low- and middle-income countries: a rapid review and case study from Burkina Faso

**DOI:** 10.3389/fgwh.2024.1511444

**Published:** 2025-01-06

**Authors:** Arthi Kozhumam, Mamadou Bountogo, Dina Goodman Palmer, Carolyn Grieg, Maxime Inghels, Sandra Agyapong-Badu, Cristina Osborne, Guy Harling, Till Bärnighausen, David Rapp, Molly Beestrum, Justine Davies, Lisa R. Hirschhorn

**Affiliations:** ^1^ Northwestern University Feinberg School of Medicine, Robert J. Havey Institute for Global Health, Chicago, IL, United States; ^2^ Centre de Recherche en Santé de Nouna, Burkina Faso, London, United Kingdom; ^3^ Institute of Applied Health Research, University of Birmingham, Birmingham, United Kingdom; ^4^ School of Sport, Exercise and Rehabilitation Sciences, University of Birmingham, Birmingham, United Kingdom; ^5^ NIHR Birmingham Biomedical Research Centre, University Hospitals Birmingham NHS Foundation Trust and University of Birmingham, Birmingham, United Kingdom; ^6^ MRC-Versus Arthritis Centre for Musculoskeletal Ageing Research, University of Birmingham, Birmingham, United Kingdom; ^7^ Lincoln International Institute for Rural Health, University of Lincoln, Lincoln, United Kingdom; ^8^ Centre Population et Développement, Université Paris Cité, Institut de Recherche Pour le Développement, Inserm, Paris, France; ^9^ University College London, London, United Kingdom; ^10^ Africa Health Research Institute, KwaZulu-Natal, South Africa; ^11^ School of Nursing & Public Health, University of KwaZulu-Natal, Durban, South Africa; ^12^ MRC/Wits Rural Public Health & Health Transitions Research Unit (Agincourt), University of the Witwatersrand, Johannesburg, South Africa; ^13^ Heidelberg Institute of Global Health (HIGH), Heidelberg University Hospital, Heidelberg University, Heidelberg, Germany; ^14^ Harvard T.H. Chan School of Public Health, Boston, MA, United States; ^15^ Global Surgical Expedition, Glen Allen, VA, United States; ^16^ Department of Urology, University of Virginia, Charlottesville, VA, United States; ^17^ Department of Global Health, Centre for Global Surgery, Stellenbosch University, Cape Town, South Africa

**Keywords:** urinary incontinence, UI, older women, LMIC (low and middle income countries), Burkina Faso

## Abstract

The prevalence of urinary incontinence (UI) in older women in low- and middle-income countries (LMICs) is not well understood. We conducted a rapid literature review to assess the burden of UI in this population and contextualize findings from a household survey of women aged 40 and older in Nouna, in northwestern Burkina Faso. The rapid review included 21 survey articles. UI prevalence for LMIC women 40 or older varied greatly (6%–80%), with differences by socio-demographics, gynecological factors (menopausal status, birth outcomes), comorbidities (age, education, obesity, diabetes, hypertension, arthritis), behaviors (smoking status) and survey location. The studies used validated tools—the International Consultation on Incontinence Urinary Incontinence Short Form (ICIQ UI-SF) was most common (*n* = 4, 19%)—and bespoke tools that have not yet been validated. In Nouna, 983 (64.5%) of 1,524 women, completed the ICIQ UI-SF. Overall UI prevalence, defined as reporting leakage at least 2–3 times a week, was 2.6% (95% CI 1.73%–3.85%), descriptively increased with age from 0.5% in 40–49 year-olds to 6.6% in those 70 and over. Of those with UI, 88.5% experienced leakage daily, and 50% reported moderate or greater interference with daily life, yet most (88.5%) had not spoken to a healthcare provider. Multivariable analysis revealed that UI was more common among women who were not currently married and decreased with higher education levels. Both the rapid review and survey highlight the burden of UI among older women in LMICs, particularly as they age beyond 60. Given UI's association with physical and mental health, it is crucial to raise awareness of its burden, improve healthcare access, and integrate routine screening into basic healthcare services. Additionally, training healthcare providers and developing culturally appropriate interventions will help address stigma and ensure effective management of UI in this vulnerable population.

## 1 Introduction

Urinary incontinence (UI) is a common chronic condition worldwide, with global prevalence estimates reported to range from 25%–40% (1). Etiologies for UI are varied and include iatrogenic (e.g., surgical injury, radiation), obstetric (e.g., fistula), traumatic, infectious (e.g., UTI), and pharmacologic. In addition, UI can commonly relate to general loss of structural urinary tract supports that occur as women age (anatomic urinary incontinence). As age is a significant risk factor for UI, the global prevalence of UI will increase as the population ages worldwide. Additional risk factors for UI among women include smoking, diabetes, traumatic childbirth, and higher body mass index (1). Urinary incontinence has a wide impact on women, with deleterious impacts to health, quality of life, mental well-being, productivity, and finances (2–4).

There is a robust understanding of UI prevalence and impact in high-income-countries (HICs). For example, UI prevalence in US women increases from 28% to 55% in women aged 30–39 vs. 80–90, respectively (5). Studies in the United States have also found that UI carries a significant health and wellbeing burden, including impairment in social life and self-confidence, self-reported ability to work, and interests in regular daily activities (2). The economic burden of UI is also high, estimated at over 65 billion USD globally per year and rising (3, 4) and systems are not in place to support affected women. For example, women with severe incontinence often have difficulty finding or paying for medications, physician visits, laundry, and other necessary products (6) as noted in multiple HICs. The resulting shame and associated isolation with withdrawal from social life and previously-enjoyed activities is associated with reduced quality of life (QoL), sleep, and general health (2). In addition, stigma is a potential barrier to women accessing UI diagnosis and management (7). These gaps in access and availability of interventions to prevent, manage and treat UI are shown to result in significant mental, emotional, and financial burdens (2).

In contrast, much less is known about the prevalence and impact of UI in low-or-middle-income countries (LMICs), including among older women, where barriers to preventative or therapeutic UI care are significant (8). UI in LMICs is understudied (8) and UI prevalence studies from LMICs focus on obstetric and iatrogenic fistula. Existing estimates of UI unrelated to the factors are limited, and vary by population studied and age (9). Addressing the direct and indirect effects of UI is challenged by limited availability and access to interventions effective in prevention, management, and treatment of UI in older women (3, 9). Understanding the burden within and across populations has been limited by the incorporation of UI into broader population-based surveys in LMICs, and that existing standardized measures have only largely been validated in HICs (8).

Expanded knowledge of burden and unmet UI needs in older populations will inform where further work is necessary for scaling effective UI prevention, diagnosis, and management interventions that are feasible, acceptable, and affordable in LMICs. This work can then lead to testing strategies at the individual, community and health system levels to get these interventions into practice.

In this article, we report the results of a rapid literature review to understand the published range of measures and burden of UI in older women in LMICs, and factors associated with UI and related health outcomes in these populations. Rapid reviews are an accelerated evidence synthesis approach used in healthcare settings to make timely, efficient decisions (10). We chose this approach due to the limited number of existing studies and resources needed for a full narrative or systematic review. Building on the literature findings, we then present a case study of cross-sectional data from Nouna, a rural region in northwest Burkina Faso, which consists of a town and 58 surrounding villages; subsistence farming is the main economic activity in the area. This combined approach aims to highlight gaps in knowledge and to inform where more work is needed to understand the prevalence of UI and care seeking in the growing group of older women in low-resource settings and support work to expand access to interventions for prevention, diagnosis, and management.

## 2 Methods

### 2.1 Rapid review

We performed a rapid review of literature on prevalence of UI in older women, defined as women aged 40 and over. Our methods were based on Tricco's third approach (11, 12). In brief, this approach includes a literature search in more than one database; search of both published and grey literature; search limited by both date and language; screening of title/abstract and decisions for inclusion reviewed by LRH. Full-text performed by one reviewer (ASK) with no risk of bias assessment and reviewed by LRH with any differences resolved through discussion.

Comprehensive literature searching was conducted across PubMed, Scopus, and grey literature using the following title and abstract keywords: “urinary incontinence,” “UI,” “low and middle income country,” “LMIC,” “older women,” “women,” and “prevalence.” We limited articles to those in English, published from 2000 onward to identify more contemporary prevalence rates. Articles were included if they directly estimated UI prevalence using an identified measure. We developed an extraction tool in Excel to capture information on population characteristics, country, measurement tool, UI prevalence, UI risk factors, and type of UI. If possible, from the data provided, we calculated the prevalence specific to older women, defined as age 40 and older, if not presented in the article.

### 2.2 Case study on UI in women age 40 and older

We conducted a secondary analysis of cross-sectional data from the second round of the CRSN Heidelberg Aging Study in the Nouna Health and Demographic Surveillance System (HDSS) area of northwest Burkina Faso (13). Detailed description of the data collection methods from the first wave of data collection in 2018 are described elsewhere (14). The follow-up wave in 2021 used the same methods with the addition of UI questions to the survey tool using the International Consultation on Incontinence Questionnaire – Urinary Incontinence Short Form (ICIQ-UI SF). Briefly, 3,033 adults were interviewed aged 40 or older, selected via random sampling within the HDSS. Potential participants were surveyed in their home, after providing written informed consent. Survey questions used in this analysis included socio-demographics, healthcare provider visits and facilities attended, depression (Patient Health Questionnaire PHQ-9), quality of life (WHO Quality of Life WHOLQOL, anxiety (Generalized Anxiety Disorder GAD-7), cognitive functioning (Community Screening Instrument for Dementia CSID) disability (WHO Disability Assessment Schedule WHODAS), and questions for derivation of frailty using the Fried frailty score (14). Questions on UI included how often participants leaked urine, how much they think they usually leak, how much leaking interferes with everyday life, and if they have spoken to a provider about the leakage. Prevalence of UI was defined as the proportion of women reporting leakage at least 2–3 times a week and impact was measured by a 4-point Likert scale for self-reported quantity of leakage and a 10-point Likert scale for self-perception of interference of UI with everyday life. The Nouna household survey and related analyses were approved by the Comite D'ethique Pour La Recherche En Sante, Burkina Faso (reference 2018-5-053) and University of Birmingham biomedical ethical review committee, reference: ERN_21-0867.

### 2.3 Analysis of study data

Analysis was conducted using Stata version 16 (StataCorp; College Station, TX). Descriptive analyses of survey questions on how often participants leak urine, how much they leak, how much leaking interferes with everyday life, and if they have spoken to a healthcare provide about leakage were conducted for women, overall and stratified by age category [40–49, 50–59, and 60+ (given limited sample size of women above age 70)]. Logistic regression was conducted to understand the association of UI status with QoL, depression, anxiety, cognitive function, and frailty, chosen based on literature, adjusting for age. Classification of categorical variables is shown in Supplementary Table S2; PHQ, GAD, and CSID scores were analyzed as categorical, with positives indicated by GAD at least 3, PHQ at least 10, and CSID at least 7.

## 3 Results

### 3.1 Rapid review

Rapid review findings are shown in Table 1. The review was conducted from May-June 2023. We found 21 survey articles with population settings including: 12 household (15, 17, 21–23, 25, 26, 31–34)^1^ including one frail population (17), two non-gynecological outpatients (18, 28), two gynecological outpatient (19, 20), and two from other populations including one elderly residential (35) and a random sample of female university staff (16). Locations included 18 LMICs (6 from the Middle East, 2 from South Asia, 3 from Southeast Asia, 4 from Africa, and 3 from South America).

**Table 1:** Findings from rapid literature review on urinary incontinence in older women from low and middle-income countries (LMICs).

Lead author	Country	Age range	Population, (*N*)	Tool used/questions asked	UI rate found	Significant associations with UI^a^	Type of incontinence if reported
Sensoy (15)	Turkey	20–80^1^	Cross-sectional study across female survey participants 20–80 (subset used here) seen in outpatient clinic, 203	ICIQ UI SF	67.5%	Hypertension, family history of UI, menopausal status, diabetes, number of deliveries (not age specified)	Stress, urge, mixed^d^
Kasiki (16)	Turkey	65+	Random sample of women 65 + attending family health centers, 1,094	Researcher-developed questionnaire	51.6%	Number of births, abortions, age at last birth, home births; BMI, urinary tract infection	Stress, urge, mixed^d^
Daneshpajooh (17)	Iran	60 +^2^	Cross-sectional cluster sampled household survey	UDI-6	79.0%	Anxiety, depression, BMI, menopause, age, diabetes	Stress, urge, mixed^d^
Najafi (18)	Iran	60+	Cross-sectional cluster sampled household survey	ICIQ UI SF	24.9%	QoL, BMI, constipation, c-section history, hypertension, use of ARBs	Stress, urge, mixed^d^
Moudi (19)	Iran	60+	Older women household survey, 300	Researcher-developed questionnaire	34.7% (104/300)	None found	Not described
Aly (20)	Egypt	60+	Females 60 + and diagnosed with frailty attending geriatrics hospital, 130	ICIQ UI SF	80.0%	Age, functional impairment, multiparity, osteoarthritis, stroke, vaginal prolapse, laxative use	19.1% mixed (urge, stress, functional)
Prabhu (21)	India	60 +^3^	Tribal village women, 58	Researcher-developed questionnaire	43.0%	Age, parity, prolonged labor, hypertension, diabetes	Not described
Khan (22)	Khan	>60^4^	Community-based cross-sectional study of post-menopausal women in urban and rural areas, 149	Researcher-developed questionnaire	46.3%	Age, rural residence, illiteracy, SES, housing, obesity, smoking, parity, UTI	20% urge, 5% stress, 22% mixed
Murukesu (23)	Malaysia	60+	Older women in community-based survey, 814	Researcher-developed questionnaire	Overall: 19.3% Urban: 16.0% Rural: 23.0%	Ethnicity, constipation, functional mobility, muscle strength	Not assessed
Masenga (24)	Tanzania	55 +^5^	Household survey of women in rural area aged 18–90, 274	UDI-6	48.5%	Parity, length of labor, first delivery at home	15% stress, 15% urge, 19% mixed
Reigota (25)	Brazil	50+	Cross-sectional population-based study of women 50+, 622	Complaint of involuntary loss of urine^b^	52.3%	Hypertension, osteoarthritis, physical activity, BMI, negative self-perception of health, limitations in daily living	13% urge, 12% stress, 27% mixed
Kessler (26)	Brazil	60+	Elderly population in urban area in private households in areas covered by basic health care services, 1,593	Researcher-developed questionnaire^c^	20.7%	Age, ethnicity, schooling	Not assessed
Sari (27)	Indonesia	60+	Elderly people attending public health centers with no serious illness or illiteracy, 351	Researcher-developed questionnaire	6.37% (21/330)	Cough, gender, education, age, lower abdominal surgery	Not described
Sumardi (28)	Indonesia	60+	Subjects enrolled from outpatient clinics, 523	Researcher-developed questionnaire	22.2%	Cough, fecal incontinence	9.4% urge, 4.8% stress, 0.4% overflow, 4% mixed, 1.5% other, 1.8% dry OAB
Ofori (29)	Ghana	60 +^6^	Adult women seeking gynecological care in tertiary facility, 55	ICIQ UI SF	25.5%	Age, cough, education	Stress, urge, mixed^d^
Akinlusi (30)	Nigeria	50 +^7^	gynecological clinic attendees, 37	Researcher-developed questionnaire	40.5%	Older age, BMI, constipation	Stress, urge, mixed^d^
Jerez-Roig (31)	Brazil	60+	Elderly 60 + individuals residing in institutions, 321	Minimum Data Set version 3.0 (assesses UI devices and toileting)	58.88% (189/321)	Race, physical inactivity, stroke, cognitive decline, mobility impairment	4% stress, 14% urge
Skaal (32)	South Africa	40+	Random sample of female staff HR working in university campus, 78	QUID	30.7% (24/78)	Age, race, obesity	Not assessed

^a^ 11 studies found association with age, 4 with education/literacy.

^b^ questionnaire used was validated in the population.

^c^ Severity measured by: In the last 30 days, how often did this happen?; In what situations do you leak urine? Because of the problem of leaking urine, do you use liners, absorbent pads, or diapers?.

^d^ percentages not reported.

^1^ subsample calculated from ages 20–80, ^2^subsample calculated from age 60+, ^3^subsample calculated from age 20+, ^4^subsample calculated from age 41+, ^5^subsample calculated from ages 18–90, ^6^subsample calculated from age 18+, ^7^subsample calculated from ages 25–67.

ICIQ UI SF, International Consultation on Incontinence Questionnaire-Urinary Incontinence Short Form; QUID, questionnaire for urinary diagnosis; UDI-6, urogenital distress inventory.

Burden of UI ranged from 6.37% (18) to 80% (33), with most articles reporting a prevalence between 20% and 40%. Among household and outpatient surveys, UI rates ranged between 33% and 37%. The lowest prevalences were in outpatient gynecologic settings (mean 33%, range 25.5–40.5%) followed by outpatient non-gynecological (mean 36.92%, range 6.3%-51.6%) and household survey populations (mean 39.9%, range 19.3%–70%). Prevalence differed between rural (higher) and urban (lower) dwelling women (23). Many, but not all articles stratified respondents by type of incontinence reported, with mixed (stress and urge) incontinence being most prevalent.

Factors associated with self-reported UI, included socio-demographics (older age, lower education), physical health [hypertension, diabetes, high body mass index (BMI), arthritis], birth outcomes (higher parity, birth location) and family history of incontinence (Supplementary Figure S1). Four studies used the ICIQ UI SF (35), with most other studies used self-developed assessment tools. No studies reported details on any cultural or linguistic translation or adaptation. We found prevalence of functional disability, depression, self-perception of health, and cognitive deficits to be higher in women with UI (25, 28). UI was associated with lower health status (15); with women with UI reporting higher rates of anxiety, depression, lower quality of life and physical impairments (31) and job/school performance (28). UI was also associated with shame (16) as well as negatively affecting physical daily activities and personal and emotional relationships (25).

The majority of articles reported that few women sought support for their UI or associated concerns. For example, Prabhu et al's healthcare seeking rate among tribal village women in India was only 14.4%, thought to be related to reduced acceptance of incontinence in the aging process and embarrassment in seeking treatment (21). Sensoy et al. among female survey participants in Turkey noted that 65% did not receive medical help, with only 57% regarding their condition as a “health problem” (15); Skaal et al. (32) noted that while women with UI working as university staff experienced lower quality of life in South Africa, less than 5% accessed mental health care or physiotherapy for their incontinence.

### 3.2 Nouna case study

Of 1,524 women, 983 (64.5%) completed questions related to UI. Approximately one-third were each aged 40–49, 50–59, 60–89, respectively. Questionnaire outcomes are detailed in Table 2.

**Table 2:** UI question responses among women in the Nouna household survey.

	Never	Two or three times a week	Once a day	Several times a day	All the time
How often do you leak urine? (*N* = 983)	957 (97.5)	3 (0.3%)	1 (0.1%)	5 (0.5%)	17 (1.7%)
Of women reporting UI (*N* = 26)					
	None	Small amount	Moderate	Large amount	
How much do you think you usually leak?	1 (3.9%)	16 (61.5%)	7 (26.9%)	2 (7.7%)	
	1–2	3–4	5–6	7–8	9–10
Overall, how much does leaking urine interfere with your everyday life? (scale 1–10) (Mean)	8 (30.8%)	5 (19.2%)	5 (19.2%)	3 (11.5%)	5 (19.2%)
	Yes	No			
Have you spoken to a healthcare provider about the leakage?	3 (11.5%)	23 (88.5%)			

Leaking urine at least once per day was reported by 2.64% (*n* = 26) of women (Table 2). UI prevalence increased with age. Rates were higher among those who were not currently married. Of the women reporting leakage, most had leakage every day (88.5%); one third (38.5%) of women with UI said that leaking urine interfered with daily life at least at a level 5 on a scale of 1–10. However most (88.5%) had not spoken to a provider about the leakage. Of those reporting UI, 50% scored positive for depression on the Patient Health Questionnaire, 30.7% scored positive for anxiety on the Generalized Anxiety Disorder scale, 73% scored positive on the Community Screening Instrument for Dementia, and 38.5% were categorized as Frail; frequency of these outcomes was more common in those with than those without UI on univariable analysis (Table 2, Supplementary Table S2).

On multivariable analysis, controlling for age, UI was significantly associated with higher depression score (OR 24.67, 95% CI 7.37–82.58) and higher Dementia score (OR 0.0047, 95% CI 0.002-0.01). It was not significantly associated with education level, marital status, quality of life, frailty, or Generalized Anxiety (Supplementary Table S1). Additional information can be found in Supplementary Material.

## 4 Discussion

The goal of our study was to understand the current prevalence of UI among older women in LMICs, and explore UI association with health status and care seeking for UI. While we found some studies describing prevalence, we found limited studies from LMICs on rates of UI among older women in the community and clinical settings. These results showed that despite the aging of populations in LMICs, UI in women in these settings is an understudied area, identifying a need for more studies. While opportunities exist, as there has been limited inclusion of UI measures in routine surveillance surveys in LMICs such as the WHO SAGE or STEPs surveys (27), advocacy is needed.

While most studies in our rapid review used the standard ICIQ UI SF tool, others used a range of tools developed by the researchers. None of the studies reviewed reported the translation and cultural adaptation of instruments used to measure UI (29), highlighting an area where work is needed to ensure that UI measurement tools have undergone cultural and linguistic translation, and psychometric testing for use in these settings (29).

In both identified literature and our case study, we found that UI prevalence increased with age, particularly among women aged 60 and older, and highest rates seen among frail populations. While rates among the older population in Nouna were similar to those reported by other studies, the present study included more younger women (age 40–60) than many of the published studies, which may have explained the lower prevalence (2.64%) overall of UI compared to 33%–37% for household surveys in other settings (24). The study in Nouna may also differ from other household surveys including the measurement tool used and population selected, for example due to cluster sampling for populations in our review that were assessed using the ICIQ-SF.

We also found that UI represents a substantial burden for women living in LMICs and is associated with lower quality of life and symptoms of poor mental health. The condition is associated with illiteracy, and low income and education status, reflecting a higher burden among more vulnerable women who may be less able to access care. This low access was found from both the literature review and our case study of low rates of care seeking, despite women reporting negative impact of UI on mental wellness and work or personal life. Literature notes that lack of awareness of UI and its burden is pervasive and is a major contributor to low health-seeking behaviors,^1^ and that existing knowledge and practices do not parallel guidelines and best practices (33). More research to provide standardized care through algorithms that can be incorporate into routine preventative care for geriatric syndromes such as UI (34), especially for women, is necessary.

In the review and in Nouna, rates of seeking care were very low, even among women with highest levels of symptoms. We were not able to determine if this was due to low availability or knowledge of health care to address UI, discomfort in discussing UI or other reasons, another area where research is needed. Given high levels of stigma around UI and symptoms of leakage (30) likely contributing to underreporting, integrating routine screening into healthcare for older women and normalizing these discussions is the first step needed for prevention and management (30). Other work will need to include building capacity among healthcare providers on interventions for prevention and management, including providing access to assistive products and devices for incontinence (30).

## 5 Limitations

Our work has several limitations. In our empirical work in Nouna, the small number of women aged 60 and older limited our ability to estimate prevalence in this group more accurately and reduced the power to detect associated conditions. Despite this, we identified associations with marital status, depression, and frailty scores. The rapid review approach, suitable for this topic where we found limited relevant literature, was less systematic and may not have captured all relevant articles. Additionally, the language restrictions in our search may have led to missed literature. We also did not focus on reports of interventions to prevent or manage UI in older women in LMICs.

## 6 Conclusions

The burden of UI among older women is growing, but studies are lacking to inform the work needed to address this growing condition and reduce associated impact on patient outcomes including mental health, function and quality of life. Further work is also needed to support the provision of accessible, affordable and acceptable interventions for the prevention, diagnosis and management of UI at individual, population, and health system levels. Efforts should also focus on reducing the associated stigma which threatens the wellbeing of the growing population of older women. The repetition of existing LMIC study methods in other countries and settings that include information on culturally sensitive approaches and linguistic adaptations, as well as with larger sample sizes and more diverse samples (more rural, more elderly, more cross-sectional studies) is needed.

## Data Availability

The data analyzed in this study is subject to the following licenses/restrictions: the datasets and analysis code for this study can be available upon reasonable request. Requests to access these datasets should be directed to lisa.hirschhorn@northwestern.edu.

## Footnotes

^1^Axena Health Commends New Study Showing the Leva System's Long-term Treatment Efficacy for Female Urinary Incontinence - Axena Health, Inc. p. 1-8. Available online at: https://axenahealth.com/2024/01/axena-health-commends-new-studyshowing-the-leva-systems-long-term-treatment-efficacy-for-female-urinaryincontinence/ (cited 2024 October 9).

## References

[1] Danforth KN Townsend MK Lifford K Curhan GC Resnick NM Grodstein F. Risk factors for urinary incontinence among middle-aged women. Am J Obstet Gynecol. (2006) 194(2):339–45. 10.1016/j.ajog.2005.07.051 16458626 PMC1363686

[2] Debus G Kästner R. Psychosomatic aspects of urinary incontinence in women. Geburtshilfe Frauenheilkd. (2015) 75(2):165–9. 10.1055/s-0034-1396257 25797959 PMC4361165

[3] Corrado B Giardulli B Polito F Aprea S Lanzano M Dodaro C. The impact of urinary incontinence on quality of life: a cross-sectional study in the metropolitan city of Naples. Geriatrics. (2020) 5(4):96. 10.3390/geriatrics5040096 33233663 PMC7709681

[4] Zillioux J Yeaman C Desai R Sharma D Balkrishnan R Rapp D. Economic impact of urinary incontinence and pelvic organ prolapse in women in Belize. IJS Glob Health. (2023) 6(5):e0271. 10.1097/GH9.0000000000000271

[5] Melville JL Katon W Delaney K Newton K. Urinary incontinence in US women: a population-based study. Arch Intern Med. (2005) 165(5):537–42. 10.1001/archinte.165.5.537 15767530

[6] Coyne KS Wein A Nicholson S Kvasz M Chen CI Milsom I. Economic burden of urgency urinary incontinence in the United States: a systematic review. J Manag Care Pharm JMCP. (2014) 20(2):130–40. 10.18553/jmcp.2014.20.2.130 24456314 PMC10437639

[7] Pintos-Díaz MZ Alonso-Blanco C Parás-Bravo P Fernández-de-las-Peñas C Paz-Zulueta M Fradejas-Sastre V Living with urinary incontinence: potential risks of women’s health? A qualitative study on the perspectives of female patients seeking care for the first time in a specialized center. Int J Environ Res Public Health. (2019) 16(19):3781. 10.3390/ijerph16193781 31597365 PMC6801418

[8] Ansari Z White S. Managing incontinence in low-and middle income-countries: a qualitative case study from Pakistan. PLoS One. (2022) 17(7):e0271617. 10.1371/journal.pone.0271617 35839232 PMC9286225

[9] Ackah M Ameyaw L Salifu MG OseiYeboah C Agyemang ASA Acquaah K Estimated burden, and associated factors of urinary incontinence among Sub-Saharan African women aged 15–100 years: a systematic review and meta-analysis. PLOS Glob Public Health. (2022) 2(6):e0000562. 10.1371/journal.pgph.0000562 36962388 PMC10021416

[10] Rapid reviews to strengthen health policy and systems: a practical guide. Available online at: https://ahpsr.who.int/publications/i/item/2017-08-10-rapid-reviews-to-strengthen-health-policy-and-systems-a-practical-guide (cited 2024 Jun 24).

[11] Kelly SE Moher D Clifford TJ. Quality of conduct and reporting in rapid reviews: an exploration of compliance with PRISMA and AMSTAR guidelines. Syst Rev. (2016) 5(1):79. 10.1186/s13643-016-0258-9 27160255 PMC4862155

[12] Tricco AC Antony J Zarin W Strifler L Ghassemi M Ivory J A scoping review of rapid review methods. BMC Med. (2015) 13(1):224. 10.1186/s12916-015-0465-6 26377409 PMC4574114

[13] Sié A Louis VR Gbangou A Müller O Niamba L Stieglbauer G The health and demographic surveillance system (HDSS) in Nouna, Burkina Faso, 1993–2007. Glob Health Action. (2010) 3:3. 10.3402/gha.v3i0.5284 PMC2940452 20847837

[14] Odland ML Payne C Witham MD Siedner MJ Bärnighausen T Bountogo M Epidemiology of multimorbidity in conditions of extreme poverty: a population-based study of older adults in rural Burkina Faso. BMJ Glob Health. (2020) 5(3):e002096. 10.1136/bmjgh-2019-002096 32337079 PMC7170422

[15] Sensoy N Dogan N Ozek B Karaaslan L. Urinary incontinence in women: prevalence rates, risk factors and impact on quality of life. Pak J Med Sci. (2013) 29(3):818–22. 10.12669/pjms.293.3404 24353635 PMC3809317

[16] Kaşıkçı M Kılıç D Avşar G Şirin M. Prevalence of urinary incontinence in older Turkish women, risk factors, and effect on activities of daily living. Arch Gerontol Geriatr. (2015) 61(2):217–23. 10.1016/j.archger.2015.06.008 26123541

[17] Daneshpajooh A Naghibzadeh-Tahami A Najafipour H Mirzaei M. Prevalence and risk factors of urinary incontinence among Iranian women. Neurourol Urodyn. (2021) 40(2):642–52. 10.1002/nau.24597 33410537

[18] Najafi Z Morowatisharifabad MA Jambarsang S Rezaeipandari H Hemayati R. Urinary incontinence and related quality of life among elderly women in Tabas, South Khorasan, Iran. BMC Urol. (2022) 22(1):214. 10.1186/s12894-022-01171-9 36587231 PMC9805688

[19] Moudi E Samadi F Hosseini SR Bijani A Ghadimi R. Urinary incontinency in elderly women and the potential risk factors: a cohort study among the elderly women of amirkola. J Babol Univ Med Sci. (2017) 19(2):14–9.

[20] Aly WW Sweed HS Mossad NA Tolba MF. Prevalence and risk factors of urinary incontinence in Frail elderly females. J Aging Res. (2020) 2020:2425945. 10.1155/2020/2425945 32399294 PMC7201653

[21] Prabhu SA Shanbhag SS. Prevalence and risk factors of urinary incontinence in women residing in a tribal area in Maharashtra, India. J Res Health Sci. (2013) 13(2):125–30. 24077468

[22] Khan S Ansari MA Vasenwala SM Mohsin Z. The influence of menopause on urinary incontinence in the women of the community: a cross-sectional study from North India. Int J Reprod Contracept Obstet Gynecol. (2017) 6(3):911–8. 10.18203/2320-1770.ijrcog20170555

[23] Murukesu RR Singh DKA Shahar S. Urinary incontinence among urban and rural community dwelling older women: prevalence, risk factors and quality of life. BMC Public Health. (2019) 19(4):529. 10.1186/s12889-019-6870-6 31196015 PMC6565537

[24] Masenga GG Shayo BC Msuya S Rasch V. Urinary incontinence and its relation to delivery circumstances: a population-based study from rural Kilimanjaro, Tanzania. PLoS One. (2019) 14(1):e0208733. 10.1371/journal.pone.0208733 30673696 PMC6343883

[25] Reigota RB Pedro AO de Souza Santos Machado V Costa-Paiva L Pinto-Neto AM. Prevalence of urinary incontinence and its association with multimorbidity in women aged 50 years or older: a population-based study. Neurourol Urodyn. (2016) 35(1):62–8. 10.1002/nau.22679 25358890

[26] Kessler M Facchini LA Soares MU Nunes BP França SM Thumé E. Prevalence of urinary incontinence among the elderly and relationship with physical and mental health indicators. Rev Bras Geriatr E Gerontol. (2018) 21:397–407. 10.1590/1981-22562018021.180015

[27] Sari W. Prevalence and associated factors of urinary incontinence among elderly in pekanbaru, Indonesia. Makara J Health Res. (2021) 25(1):27–33. Available online at: https://scholarhub.ui.ac.id/mjhr/vol25/iss1/5

[28] Sumardi R Mochtar CA Junizaf Santoso BI Setiati S Nuhonni SA, Prevalence of urinary incontinence, risk factors and its impact: multivariate analysis from Indonesian nationwide survey. Acta Medica Indones. (2014) 46(3):175–82. 25348179

[29] Ofori AA Osarfo J Agbeno EK Azanu WK Opare-Addo HS. Prevalence and determinants of non-fistulous urinary incontinence among Ghanaian women seeking gynaecologic care at a teaching hospital. PLoS One. (2020) 15(8):e0237518. 10.1371/journal.pone.0237518 32810136 PMC7433879

[30] Akinlusi F Oshodi Y Seriki B Olalere FD Kuye Kuku T. Female urinary incontinence: prevalence, risk factors and impact on the quality of life of gynecological clinic attendees in Lagos, Nigeria. Nepal J Obstet Gynaecol. (2020) 15:31–8. 10.3126/njog.v15i1.29338

[31] Jerez-Roig J Santos MM Souza DLB Amaral FLJS Lima KC. Prevalence of urinary incontinence and associated factors in nursing home residents. Neurourol Urodyn. (2016) 35(1):102–7. 10.1002/nau.22675 25307780

[32] Skaal L Mashola MK. The prevalence of urinary incontinence and its impact on quality of life among the university female staff in South Africa. South Afr J Physiother. (2011) 67(2):45–9.

[33] Janse van Vuuren AC van Rensburg JA Hanekom S. Practitioner’s knowledge, attitudes, beliefs and practices towards urinary incontinence. South Afr J Physiother. (1860) 79(1):485–99. 10.4102/sajp.v79i1.1860 PMC10319925 37415853

[34] Geriatric Syndromes: Clinical, Research, and Policy Implications of a Core Geriatric Concept - Inouye - 2007 - Journal of the American Geriatrics Society—Wiley Online Library. Available online at: https://agsjournals.onlinelibrary.wiley.com/doi/full/10.1111/j.1532-5415.2007.01156.x (cited 2024 October 9). 10.1111/j.1532-5415.2007.01156.x PMC2409147 17493201

[35] ICIQ-UI SF | ICIQ. Available online at: https://iciq.net/iciq-ui-sf (cited 2024 Jun 17).

