# Supplementary material for: Urinary incontinence (UI) in older women in low- and middle-income countries: a rapid review and case study from Burkina Faso
Source: Front Glob Womens Health. 2025 Jan 6;5:1511444. doi: 10.3389/fgwh.2024.1511444 (PMC11743521; doi:10.3389/fgwh.2024.1511444)
Supplement: Supplementary file 1 [file Supplementaryfile1.docx]

Supplementary Material

## Supplementary Figures


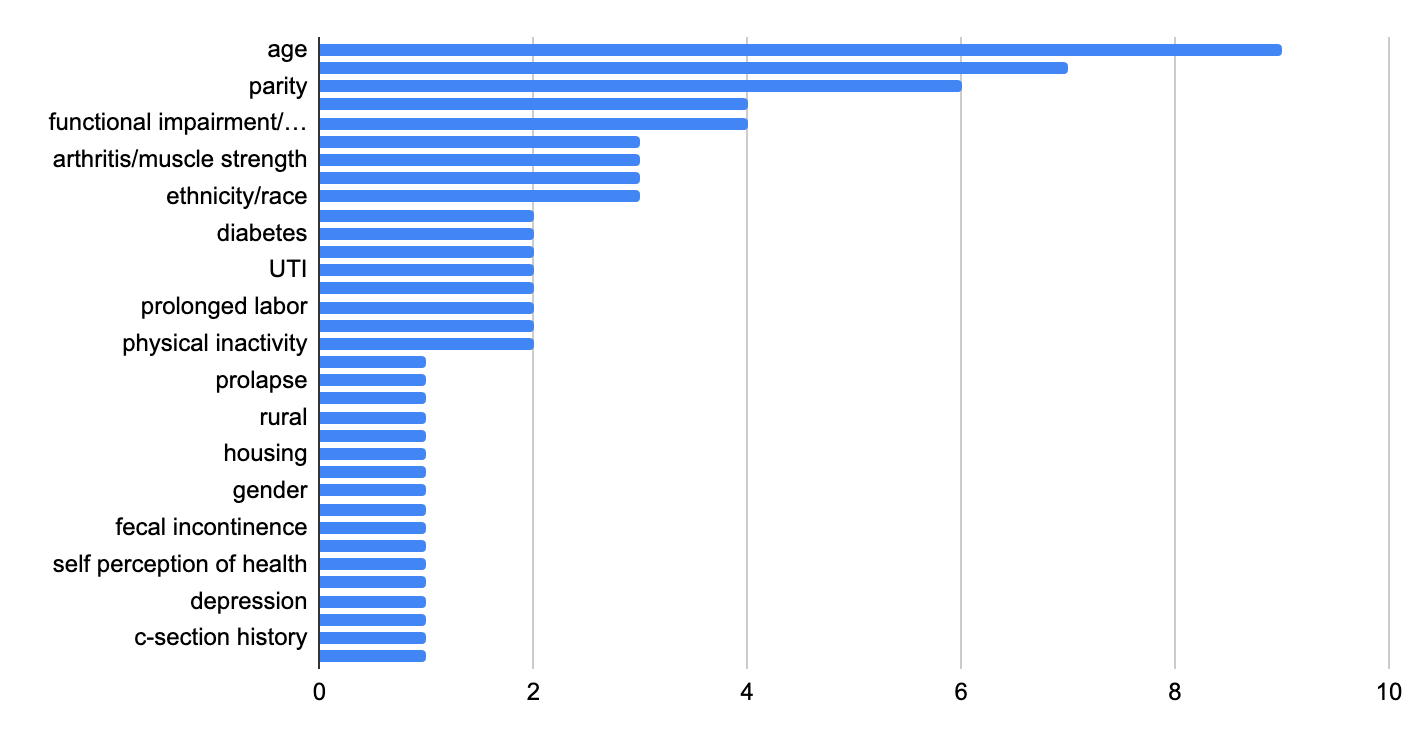


**Supplementary Figure 1.** Variables noted as associated with UI in rapid review articles; most frequently noted were age, parity, and functional impairment.

## Supplementary Tables

**Supplementary Table 1.** Logistic regression output. CSID and PHQ scores found to be significantly associated with UI.

| Variable | OR | 95% CI | p-value |
| --- | --- | --- | --- |
| age | .98 | .95 1.01 | 0.25 |
| education | .81 | .47 1.38 | 0.44 |
| marital status  -married  -separated  -divorced  -widowed  -cohabitating | 0.24  0.19  0.19  0.47  0.64 | 0.0004 131.23  0.0001. 234.27  0.0001. 270.20  0.0008. 260.77  0.0010. 428.17 | 0.659  0.648  0.656  0.814  0.894 |
| QoL | .99 | .96 1.01 | 0.34 |
| PHQ (positive) | **24.80** | 7.38 83.33 | 0.00 |
| GAD (positive) | **2.56** | .83 7.90 | 0.10 |
| CSID (positive) | **.005** | .002 .01 | 0.00 |

**Supplementary Table 2.** Characteristics of women in sample with and without UI

|  | **No UI** | **UI** |
| --- | --- | --- |
| **Age** |  |  |
| 40-49 | 356 | 1 |
| 50-59 | 297 | 7 |
| 60-69 | 196 | 12 |
| 70+ | 107 | 6 |
| **Education** |  |  |
| No formal schooling | 856 | 26 |
| Less than primary | 56 | 0 |
| Primary complete | 25 | 0 |
| Some secondary | 13 | 0 |
| Secondary complete | 5 | 0 |
| Some high school | 1 | 0 |
| High school complete | 1 | 0 |
| **Marital status** |  |  |
| Never married | 5 | 0 |
| Currently married | 569 | 8 |
| Separated | 13 | 0 |
| Divorced | 6 | 1 |
| Widowed/Cohabitating | 363 | 17 |
| **PHQ** |  |  |
| Negative | 856 | 13 |
| Positive | 101 | 13 |
| **GAD** |  |  |
| Negative | 853 | 18 |
| Positive | 104 | 8 |
| **CSID** |  |  |
| Negative | 24 | 7 |
| Positive | 933 | 19 |
| **Fried Frailty** |  |  |
| Robust | 420 | 4 |
| Pre-frail | 458 | 11 |
| Frail | 65 | 10 |
